# Antibodies to *Pseudogymnoascus destructans* are not sufficient for protection against white-nose syndrome

**DOI:** 10.1002/ece3.1502

**Published:** 2015-05-11

**Authors:** Joseph S Johnson, DeeAnn M Reeder, Thomas M Lilley, Gábor Á Czirják, Christian C Voigt, James W McMichael, Melissa B Meierhofer, Christopher W Seery, Shayne S Lumadue, Alexander J Altmann, Michael O Toro, Kenneth A Field

**Affiliations:** 1 Department of Biology, Bucknell UniversityLewisburg, Pennsylvania, 17837; 2 Department of Biology, University of TurkuTurku, Finland; 3 Leibniz Institute for Zoo and Wildlife ResearchBerlin, Germany

**Keywords:** Antibody-mediated immunity, antifungal immunity, *Corynorhinus*, disease ecology, *Eptesicus*, hibernation, *Myotis*, *Perimyotis*, wildlife disease, WNS

## Abstract

White-nose syndrome (WNS) is a fungal disease caused by *Pseudogymnoascus destructans* (*Pd*) that affects bats during hibernation. Although millions of bats have died from WNS in North America, mass mortality has not been observed among European bats infected by the fungus, leading to the suggestion that bats in Europe are immune. We tested the hypothesis that an antibody-mediated immune response can provide protection against WNS by quantifying antibodies reactive to *Pd* in blood samples from seven species of free-ranging bats in North America and two free-ranging species in Europe. We also quantified antibodies in blood samples from little brown myotis (*Myotis lucifugus*) that were part of a captive colony that we injected with live *Pd* spores mixed with adjuvant, as well as individuals surviving a captive *Pd* infection trial. Seroprevalence of antibodies against *Pd*, as well as antibody titers, was greater among little brown myotis than among four other species of cave-hibernating bats in North America, including species with markedly lower WNS mortality rates. Among little brown myotis, the greatest titers occurred in populations occupying regions with longer histories of WNS, where bats lacked secondary symptoms of WNS. We detected antibodies cross-reactive with *Pd* among little brown myotis naïve to the fungus. We observed high titers among captive little brown myotis injected with *Pd*. We did not detect antibodies against *Pd* in *Pd*-infected European bats during winter, and titers during the active season were lower than among little brown myotis. These results show that antibody-mediated immunity cannot explain survival of European bats infected with *Pd* and that little brown myotis respond differently to *Pd* than species with higher WNS survival rates. Although it appears that some species of bats in North America may be developing resistance to WNS, an antibody-mediated immune response does not provide an explanation for these remnant populations.

## Introduction

White-nose syndrome (WNS) is a fungal disease responsible for precipitous declines in bat populations in North America (Lorch et al. 2011; Blehert 2012; Reeder and Moore 2013; Frick et al. 2015). Since its discovery in New York in 2006, millions of bats have died from WNS, with mortality continuing at an alarming rate as the disease spreads across the United States and Canada (Coleman and Reichard 2014; Frick et al. 2015). Population declines in excess of 90% have been estimated for several species, leading to predictions of regional and species-level extinctions in the near future (Frick et al. 2010, 2015; Turner et al. 2011).

WNS is caused by the psychrophilic fungus *Pseudogymnoascus destructans* (*Pd*), originally classified as *Geomyces destructans* in 2009 (Gargas et al. 2009; Minnis and Lindner 2013). Suitable temperatures for *Pd* growth overlap with temperatures inside bat hibernacula (Webb et al. 1996; Humphries et al. 2002; Verant et al. 2012), allowing the fungus to grow in the winter habitat of many bat species. *Pd* invades the dermis and epidermis of bats while they hibernate (Blehert et al. 2009), and infection is likely facilitated by the reduction in immune function typical of hibernation (Bouma et al. 2010a). Fungal colonization causes fatal disruptions in behavior (Brownlee-Bouboulis and Reeder 2013; Wilcox et al. 2014) and physiology (Verant et al. 2014), including energy and water balance (Cryan et al. 2010, 2013). The critical disruption in energy balance is illustrated by the hibernation ecology of little brown myotis (*Myotis lucifugus*) that, when affected by WNS, exhibit an increase in frequency of arousals from hibernation, depleting fat reserves necessary to survive winter (Reeder et al. 2012; Warnecke et al. 2012). Little brown myotis suffering from WNS are also more dehydrated than healthy bats, suggesting evaporative water loss could at least partially explain this increase in arousals (Cryan et al. 2013).

*Pd* is not native to North America; it is believed to have been introduced from Europe, where it is widespread (Puechmaille et al. 2011; Warnecke et al. 2012; Wibbelt et al. 2013). European isolates of *Pd* cause mortality in North American bats (Warnecke et al. 2012), but European bats infected with *Pd* do not appear to have the same pathology (Wibbelt et al. 2013). The discovery of *Pd* growing on European bats during hibernation led to the suggestion that European bats may be immune to WNS (Puechmaille et al. 2010). However, some mammal immune responses have been shown to be suppressed during hibernation (Bouma et al. 2010a). Hibernating bats, therefore, likely have a limited ability to mount an immune response to *Pd* during the period of active infection. The reduction of immune function during hibernation should not be interpreted to mean that a protective immune response is not possible, however, as both cell- and antibody-mediated (humoral) immune responses can occur during hibernation (Maniero 2002; Bouma et al. 2013). While antibody-mediated immune responses to fungi can help clear infections, they can also lead to tolerance of chronic infection (Casadevall and Pirofski 2012a,b; Wüthrich et al. 2012). Thus, the alternative hypothesis that activation of an immune response during winter contributes to pathology and mortality among North American species must also be considered.

The capacity to mount an immune response to *Pd* may differ among bat species. For example, the little brown myotis is a small bat (6–14 g) that exhibits relatively long periods of torpor between periodic arousals from hibernation compared to larger species such as the big brown bat (*Eptesicus fuscus*) (Brack and Twente 1985; Twente et al. 1985; Reeder et al. 2012). This greater frequency of arousals in big brown bats may result in greater immune competence and ability to respond to *Pd* during winter, possibly explaining the lower WNS mortality rates reported for big brown bats (Turner et al. 2011; Frank et al. 2014).

The purpose of our study was to examine the role of antibody-mediated immune responses to *Pd* in captive and free-ranging bats. We hypothesized that captive little brown myotis exposed to *Pd* during hibernation would have greater antibody titers in the spring than bats not exposed to the pathogen. A secondary goal of our captive study was to determine whether seroprevalence and titers could be experimentally increased by injecting captive little brown myotis with live *Pd*. Among free-ranging bats, our goals were to determine whether free-ranging bats in North America and Europe produce antibodies against *Pd*, when antibody levels peak, and whether antibody production varies among species and geographic regions. We also tested whether antibody seroprevalence, defined as a blood sample with detectable antibodies reactive to *Pd*, and titer are correlates of WNS survival. We hypothesized that European species would exhibit greater seroprevalence and titers than any North American species. Within North America, we hypothesized that populations inhabiting regions with longer histories of WNS would exhibit the greatest seroprevalence and titers, and that titers would peak shortly after the end of hibernation. Finally, we hypothesized that North American species with higher WNS survival rates and winter ecologies favoring more frequent arousals from hibernation would exhibit the greatest seroprevalence and titers.

## Materials and Methods

This study was carried out on bats from nonendangered species in strict accordance with the recommendations in the Guide for the Care and Use of Laboratory Animals of the National Institutes of Health. All methods were approved by the Institutional Animal Care and Use Committee at Bucknell University (protocol DMR-016), the Animal Ethics Committee of the University of Turku (license number ESAVI/3221/04.10.07/2013), and the Leibniz Institute for Zoo and Wildlife Research Berlin.

### Captive studies

We established a captive population of little brown myotis naïve to *Pd* in 2013. We captured bats in mist-nets (Avinet, Inc., Dryden, NY) placed outside known WNS-free roosts in Montana, USA, and transported to our laboratory at Bucknell University in Pennsylvania, USA. Following arrival, 26 bats were randomly selected for an immunization trial to determine whether anti-*Pd* antibody titers could be boosted through injection of *Pd* preparations. Ten of these bats were given intraperitoneal injections of 6 × 10^6^ live *Pd* cells suspended in 0.1 mL of phosphate buffered saline (PBS) emulsified in 0.1 mL of the Novartis adjuvant MF59, which has been successfully used in generating protective antifungal immunity in mice (Torosantucci et al. 2005). As a control, 16 bats that were housed separately were given intraperitoneal injections containing only 0.2 mL PBS. Bats were given intraperitoneal booster injections of either 0.2 mL PBS (control bats) or 6 × 10^6^ live *Pd* cells suspended in 0.2 mL of PBS (immunized bats) at 1 and 3 weeks following initial injections. We collected plasma samples from all 26 bats 6 weeks following initial injections. We collected blood into heparinized glass microhematocrit capillary tubes (Kimball Chase Life Science, Vineland, NJ) after puncturing a vein in the uropatagium using a 27.5-gauge sterile needle (Reeder and Widmaier 2009). Capillary tubes were immediately centrifuged to separate plasma from blood cells, and plasma was stored at −80°C.

For a separate captive infection experiment, we collected 147 little brown myotis naïve to *Pd* from WNS-free hibernacula in Michigan and Illinois in November of 2012 and brought them back to our laboratory (Johnson et al. 2014). Bats were either cutaneously inoculated with *Pd* (*n* = 118) or sham inoculated (*n* = 29) with PBS, and hibernated for 5 months in captivity. *Pd*-inoculated bats were hibernated in a separate chamber from control bats, and the two groups were housed separately upon arousal from hibernation. *Pd* used for inoculations was obtained from an isolate harvested from a little brown myotis in Pennsylvania in 2010. In order to determine whether or not surviving WNS results in the generation of antibodies against *Pd*, we collected nonterminal blood samples from 63 surviving bats (control: *n* = 19; *Pd* inoculated: *n* = 44) at 2, 6, and 10 weeks following the end of hibernation. Blood was collected and stored as described above.

### Field studies

We captured bats at night using mist-nets, or captured roosting bats by hand during the day in order to collect nonterminal blood samples for our analysis. We collected blood as described above, except that plasma was stored on dry ice until transferred to our laboratory, where it was stored at −80°C. Blood was only collected from adult bats. In addition to collecting blood, we scored the wings for damage considered to be a secondary symptom of WNS (Reichard and Kunz 2009) and swabbed the wings and muzzles to later determine the number of *Pd* cells present (Johnson et al. 2014).

We collected blood from free-ranging little brown myotis, northern myotis (*M. septentrionalis*), big brown bats, tri-colored bats (*Perimyotis subflavus*), Rafinesque's big-eared bats (*Corynorhinus rafinesquii*), evening bats (*Nycticeius humeralis*), and eastern red bats (*Lasiurus borealis*) captured at Mammoth Cave National Park, Kentucky, USA (see Table S1). Eastern red bats were the only species in our study not known to hibernate in caves. Samples were collected from late May through early June (spring) of 2013 and 2014, and during late July (summer) of 2013. Samples were also collected from little brown myotis during the spring in New York, Pennsylvania, and Montana (see Table S1). We also collected samples from Daubenton's myotis (*M. daubentonii*) in southwestern Finland during the spring and summer of 2013. Bats in Finland did not show any sign of wing damage typical of WNS survivors in North America (Meteyer et al. 2009; Reichard and Kunz 2009).

We collected terminal blood samples from little brown myotis and tri-colored bats collected from two WNS-positive caves in Kentucky, USA, in March (winter) 2014. These bats were selected for sampling based upon the presence of visible fungus. We swabbed the muzzles and wings of bats to confirm the presence of *Pd* by qPCR (Johnson et al. 2014). Bats were aroused for ≥1 h before being euthanized using isoflurane followed by decapitation. We also collected terminal winter samples from a European species, greater mouse-eared myotis (*M. myotis*), with (*n* = 7) and without (*n* = 7) visible fungal growth, presumed to be *Pd*, from three hibernacula in Northern Bavaria, Germany, during March 2012. Bats were immediately euthanized upon collection and were not aroused prior to collection of blood samples. Blood was processed and stored as described above.

### Measuring anti-*Pd* antibody titers

Plasma antibody titers were measured using an enzyme-linked immunosorbent assay (ELISA) in U-bottom 96-well plates with 100,000 formalin-fixed *Pd* conidia suspended in 200 *μ*L PBS containing 0.05% Tween-20 (PBST) and 1% bovine serum albumin (PBST+BSA). The concentration of *Pd* cells was determined using a hemocytometer. Plates were spun in a centrifuge at 600 *g* for 5 min, and the supernatant discarded. We then added 50 *μ*L of bat plasma diluted in PBST+BSA at concentrations of 1:100 and 1:1000 to wells and incubated on a plate shaker for 1 h at 20–24°C. Cells were then washed twice with PBS before adding 50 *μ*L of biotinylated protein A/G (BioVision, Inc., Milpitas, CA) diluted in PBST+BSA at a concentration of 1:10,000 and incubated on a plate shaker for 1 h at 20–24°C. Cells were then washed twice with PBS before adding 50 *μ*L streptavidin peroxidase polymer, ultrasensitive (Sigma-Aldrich, St. Louis, MO) diluted in PBST+BSA at a concentration of 1:2000 and incubated for 30 min on a plate shaker at 20–24°C. Cells were then washed three times with PBS before incubating with 100 *μ*L tetramethylbenzidine (eBioscience, San Deigo, CA). The reaction was terminated after 15 min using 10 *μ*L of 10% trichloroacetic acid. Absorbance was measured at 450 nm on a microplate spectrophotometer (BioTek *μ*Quant, Winooski, VT). Background absorbance was subtracted from all samples using negative control wells that received PBST+BSA instead of plasma. Serum from a rabbit immunized and boosted with lyophilized *Pd* (LAMPIRE Biological Laboratories, Pipersville, PA) was used for a positive control and to validate the assay. Samples with absorbance <0.1 units above background (3 standard deviations) were assigned a titer of 10 and considered not-detectable for anti-*Pd* antibodies. For the remaining samples, the bat was considered to be seropositive and titer was calculated by multiplying the absorbance by the dilution. The titer from the sample diluted 1:100 was used unless both 1:100 and 1:1000 titers were above 50, and then, the largest titer of the two concentrations was assigned. Because samples were analyzed on different days, positive titers were standardized across assays using the average absorbance value of triplicate samples of the rabbit anti-*Pd* positive control at a dilution of 1:1000. All plasma samples were measured on two separate days and produced similar results on each day after standardization.

### Statistical analysis

We used chi-square tests to compare seroprevalence (the proportion of samples with detectable antibodies against *Pd*) between captive little brown myotis cutaneously inoculated with *Pd* and sham inoculated with PBS. Bats testing positive for anti-*Pd* antibodies at any of the three time points were considered positive for antibodies for this comparison. We tested our hypothesis that titers would peak shortly after hibernation using a longitudinal nonparametric alternative to a repeated measures analysis, Friedman's test, because titer data could not be transformed to meet the assumptions of parametric tests. We used Wilcoxon rank-sum tests as a method of means comparisons between time points. We also used Wilcoxon rank-sum tests to compare antibody titers between the two groups at each time point to test the hypothesis that titers would be greatest among little brown myotis inoculated with *Pd*. Similarly, we compared seroprevalence between little brown myotis immunized with live *Pd* and bats sham-injected using chi-square tests and compared titers between the two groups using a Wilcoxon rank-sum test.

We used chi-square tests to compare seroprevalence and Kruskal–Wallis tests to compare antibody titers to test each of our hypotheses pertaining to wild populations. When appropriate, means comparisons were made using a Wilcoxon rank-sum test or 2 × 2 chi-square tests with a sequential Bonferroni–Holm correction for each pair of treatments. To test the hypothesis that antibody seroprevalence and titers would be greatest in populations with longer histories of WNS, we compared little brown myotis sampled during spring in Montana (no known WNS history) to populations in Kentucky (WNS-positive since 2011), Pennsylvania (WNS-positive since 2008), and New York (WNS-positive since 2006). Data collected in Kentucky during the spring seasons of 2013 and 2014 were combined following verification that seroprevalence and titer did not vary between years (Table S2). We also compared seasonal differences in titers for species sampled at the same location at different times of year. To test the hypothesis that European species would exhibit greater seroprevalence and titer, we compared greater mouse-eared myotis samples to samples from North American species also collected during hibernation and compared Daubenton's myotis to little brown myotis populations in New York, our North American sampling location with the longest history of WNS, also sampled during spring.

Changes in *Pd* loads quantified by PCR on free-ranging bats captured in Kentucky between 2013 and 2014 were compared for each species individually using a Wilcoxon rank-sum test. Kruskal–Wallis tests were used to compare *Pd* loads among species captured in Kentucky and to compare *Pd* loads on little brown myotis captured in Kentucky, Pennsylvania, and New York. Because *Pd* loads in several species differed between years, comparison among species captured in Kentucky was limited to species captured in 2014, and only little brown myotis captured in 2014 were compared to bats captured in New York and Pennsylvania. For illustrating comparisons across time and between species, data for some little brown myotis groups are found in more than one figure.

## Results

**Figure 1:**
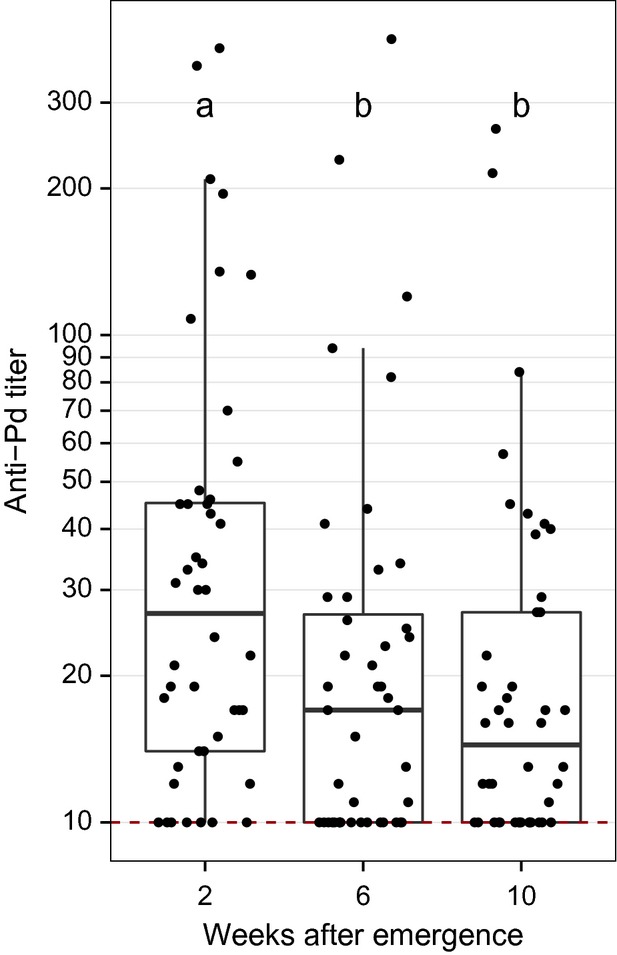
Comparison of anti-*Pd* antibody titers in 44 little brown myotis (*Myotis lucifugus*) collected from *Pd*-negative caves cutaneously inoculated with *Pd* and hibernated in captivity for 5 months. Anti-*Pd* titers were highest shortly after the end of hibernation and then declined. Data are presented with quartiles, median (black line), and whiskers that represent 1.5 times the interquartile range. Titers ≤ 10 are considered negative for *Pd* reactive antibodies. Titers differed significantly between weeks not sharing common letters (*P* < 0.05).

**Figure 2:**
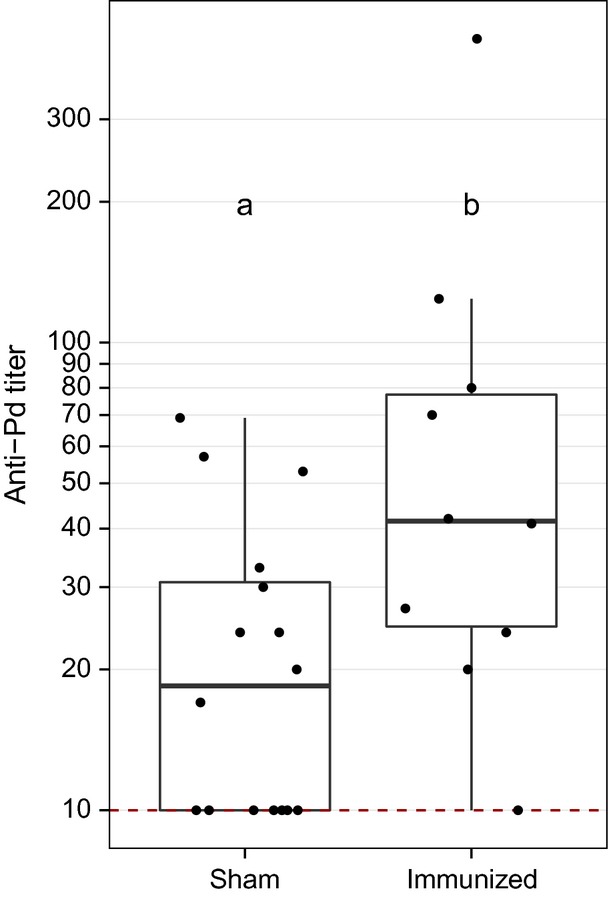
Comparison of anti-*Pd* antibody titers in 26 little brown myotis (*Myotis lucifugus*) collected from outside the WNS-affected region of North America and immunized with *Pd* emulsified with adjuvant or PBS (sham). Anti-*Pd* titers were greater in immunized bats, as denoted by different letters (*P* < 0.05). Data are presented as quartiles, median (black line), and whiskers that represent 1.5 times the interquartile range. Titers ≤ 10 are considered negative for *Pd* reactive antibodies.

To test whether little brown myotis mount an antibody response to *Pd* when infected with the fungus, we compared anti-*Pd* antibody levels in captive bats previously infected with *Pd* (*n* = 44) to uninfected conspecifics (*n* = 44). Fifty-seven of 63 (90%) samples from captive little brown myotis collected from WNS-negative caves tested positive for anti-*Pd* antibodies upon arousal from hibernation. There was no difference in seroprevalence (*χ*^2^ = 0.08, df = 1, *P* = 0.77) or titer (week 2: *Z* = −0.63, *P* = 0.53; week 6: *Z* = −1.40, *P* = 0.16; week 10: *Z* = −0.05, *P* = 0.96) between groups cutaneously inoculated with *Pd* or sham inoculated with PBS. Anti-*Pd* antibody titers differed across weeks among inoculated bats (*χ*^2^ = 15.6, df = 2, *P* < 0.001; Fig.1), with titers at week 2 being significantly greater than at week 6 (*χ*^2^ = 9.8, df = 2, *P* = 0.002) and week 10 (*χ*^2^ = 10.5, df = 2, *P* = 0.001). To determine whether injection with *Pd* could increase anti-*Pd* antibody levels, we injected little brown myotis with live *Pd* mixed with an adjuvant. Compared to bats that were injected with PBS, injected bats exhibited significantly greater anti-*Pd* antibody titers (Z = −2.3, *P* = 0.02), but not seroprevalence (*χ*^2^ = 3.3, df = 1, *P* = 0.07) (Fig.2).

**Figure 3:**
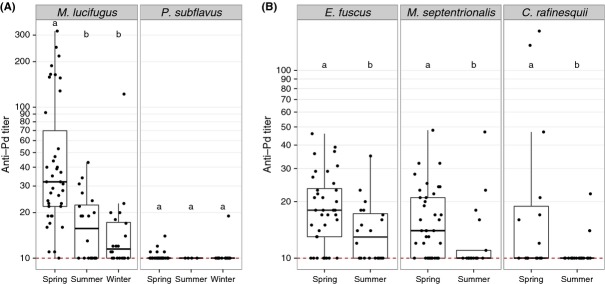
Comparison of anti-*Pd* antibody titers in five free-ranging bat species sampled in Kentucky, USA. (A) Anti-*Pd* titers were greater in little brown myotis (*Myotis lucifugus*) than in tri-colored bats (*Perimyotis subflavus*) (*P* < 0.05) and were greatest during the spring in little brown myotis. (B) Big brown bats (*Eptesicus fuscus*), northern long-eared myotis (*M. septentrionalis*), and Rafinesque's big-eared bats (*Corynorhinus rafinesquii*) had greater anti-*Pd* titers in spring than in summer, and all had lower titers than little brown bats in the spring (*P* < 0.05). Data are presented as quartiles, median (black line), and whiskers that represent 1.5 times the interquartile range. Titers ≤ 10 are considered negative for *Pd* reactive antibodies. Within each species, titers differed significantly between seasons not sharing common letters (*P* < 0.05).

Next, we determined whether different species have variable anti-*Pd* antibody levels throughout the year. Comparing little brown myotis and tri-colored bats sampled during the spring, summer, and winter in Kentucky (Fig.3A), we found little brown myotis exhibited greater seroprevalence (*χ*^2^ = 18.0, df = 2, *P* < 0.001, Table S3) and titers (*χ*^2^ = 29.5, df = 2, *P* < 0.001, see Table S3) during spring than during winter or summer, but no significant seasonal differences were detected among tri-colored bats (seroprevalence: *χ*^2^ = 1.3, df = 2, *P* = 0.52; titer: *χ*^2^ = 1.2, df = 2, *P* = 0.56). Among three species sampled during the spring and summer (Fig.3B), northern myotis exhibited greater seroprevalence (*χ*^2^ = 8.0, df = 1, *P* = 0.005) and titer (*Z* = −2.5, *P* = 0.01) during spring compared to summer, as did Rafinesque's big-eared bats (*χ*^2^ = 4.2, df = 1, *P* = 0.04; titer: *Z* = −2.2, *P* = 0.03). Big brown bats exhibited greater titers (*Z* = −2.4, *P* = 0.02), but not seroprevalence (*χ*^2^ = 3.2, df = 1, *P* = 0.08) during spring. *Pd* loads varied among species sampled in Kentucky in 2014 (*χ*^2^ = 30.0, df = 4, *P* < 0.001), with little brown myotis having the greatest *Pd* loads (Table S4). Comparing anti-*Pd* antibodies in North American species sampled in the same location (Kentucky) at the same time (spring), seroprevalence (*χ*^2^ = 60.8, df = 4, *P* < 0.001) and titer (*χ*^2^ = 74.03, df = 4, *P* < 0.001) significantly varied, with little brown myotis exhibiting higher seroprevalence and titer compared to all other species (Table S5). Eastern red bats and evening bats were not included in comparisons due to sample size but are reported in Table S1. Antibodies against *Pd* were never detected among red bats.

**Figure 4:**
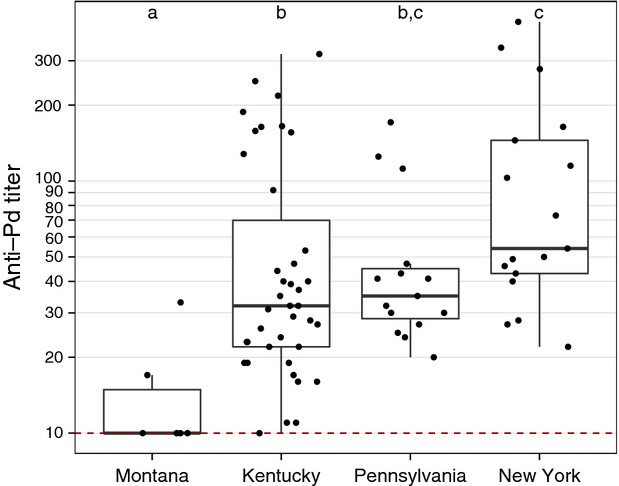
Comparison of anti-*Pd* antibody titers determined for little brown myotis (*Myotis lucifugus*) captured in spring across North America. Anti-*Pd* titers are lowest, but still detectable, in Montana (WNS-negative), followed by Kentucky (WNS-positive since 2011), then Pennsylvania (WNS-positive since 2008), and the highest titers were found in New York (WNS-positive since 2006). Data are presented as quartiles, median (black line), and whiskers that represent 1.5 times the interquartile range. Titers ≤ 10 are considered negative for *Pd* reactive antibodies. Titers differed significantly between locations not sharing common letters (*P* < 0.05).

To determine whether anti-*Pd* titers correlate with length of exposure of a population to WNS, we measured anti-*Pd* antibody levels in four North American populations of little brown myotis during the spring. Seroprevalence (*χ*^2^ = 40.0, df = 3, *P* < 0.001) and titer (*χ*^2^ = 17.8, df = 3, *P* < 0.001) significantly varied, with little brown myotis exhibiting higher titers in New York than in all states except Pennsylvania (Fig.4, Table S6). Among little brown myotis sampled in New York and Pennsylvania, only one bat had noticeable wing damage, receiving a Reichard score of 1 (Reichard and Kunz 2009). The extent of *Pd* infection appeared to increase among bats in Kentucky between 2013 and 2014. Seven of 20 (35%) of little brown myotis captured in Kentucky in 2013 had a wing damage score of 1, with the remaining 65% bats exhibiting no damage, but 61% (*n* = 11) of bats sampled at the same location in 2014 had moderate (damage index = 2) or severe damage (value = 3). Furthermore, the median number of *Pd* cells (genomic equivalents) detected by qPCR on little brown myotis (*Z* = −4.3, *P* < 0.001), northern myotis (*Z* = −4.3, *P* < 0.001), big brown bats (*Z* = −4.9, *P* < 0.001), and tri-colored bats (*Z* = −3.2, *P* = 0.02) increased between 2013 and 2014. We also detected greater *Pd* loads on little brown myotis captured in Kentucky during 2014 than in New York or Pennsylvania (Table S7).

**Figure 5:**
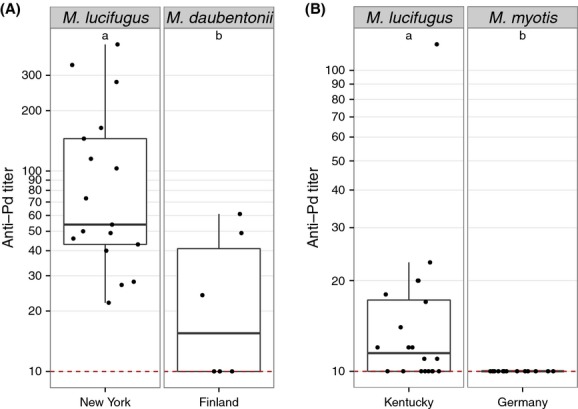
Comparison of anti-*Pd* antibody titers in free-ranging bat species in North America and Europe. (A) Anti-*Pd* titers were greater in little brown myotis (*Myotis lucifugus*) in New York, USA, than in Daubenton's myotis (*M. daubentonii*) in Finland during spring. (B) Anti-*Pd* titers were greater in little brown myotis hibernating in Kentucky, USA, than in Daubenton's myotis hibernating in Germany. Data are presented as quartiles, median (black line), and whiskers that represent 1.5 times the interquartile range. Titers ≤ 10 are considered negative for *Pd* reactive antibodies. Titers differed significantly between species not sharing common letters within each panel (*P* < 0.05).

To determine whether anti-*Pd* antibody levels differ between North American and European species, we compared antibody levels in two species of European bats to *M. lucifugus* in Kentucky and New York. Winter seroprevalence (*χ*^2^ = 20.2, df = 2, *P* < 0.001, Table S8) and titers (*χ*^2^ = 18.8, df = 2, *P* < 0.001, Table S8) differed between North American and European species, with no antibodies against *Pd* detected among greater mouse-eared myotis in Germany (Fig.5, Table S8). We were able to detect anti-*Pd* antibodies among Daubenton's myotis sampled during the spring in Finland, but titers were significantly lower (*Z* = −2.4, *P* = 0.02) and were less likely to be seropositive (*χ*^2^ = 9.8, df = 1, *P* = 0.002) than little brown myotis sampled in New York during the same time period (Fig.5). Summer samples from Daubenton's myotis were not included in analyses due to low sample size, but are reported in Table S1.

## Discussion

We present the first evidence of an antibody-mediated immune response to *Pd* in bats. Contrary to our prediction, we did not observe greater seroprevalence or titers of anti-*Pd* antibodies in European than in North American species, and notably, we did not detect antibodies against *Pd* among greater mouse-eared myotis both infected and uninfected with *Pd* during hibernation. Similarly, anti-*Pd* antibody levels in a second European species, Daubenton's myotis, were also found to be lower than in little brown myotis of North America, although their *Pd* exposure status was unknown. Thus, bats in Europe, which have presumably lived with *Pd* for thousands of years, have lower antibody titers than species in North America that have only recently become exposed. These data provide strong evidence that an antibody-mediated immune response is not the mechanism of survival among European bats infected by *Pd*. This conclusion is further supported by our finding that little brown myotis, a species with WNS mortality rates >90% (Turner et al. 2011), have higher anti-*Pd* antibody titers than any North American or European species, while some species with lower mortality rates had lower titers. Furthermore, antibodies reactive against *Pd* were identified in little brown myotis that had never been exposed to the fungus. Were antibodies involved in defense against *Pd*, one would expect that little brown myotis would experience higher survival rates given the prevalence of antibodies in naïve populations.

Consistent with our predictions, however, populations of little brown myotis inhabiting New York and Pennsylvania, where WNS has been present since 2006 and 2008, respectively, had greater seroprevalence and titers than naïve and more recently exposed populations. Little brown myotis in New York and Pennsylvania also exhibited less severe wing damage and had fewer *Pd* cells on their skin compared little brown myotis from Kentucky, where WNS arrived more recently. The presence of *Pd* on the skin of bats in Pennsylvania and New York, along with the presence of *Pd* in hibernacula across both states, strongly suggests that these bats had survived a winter exposed to *Pd*, yet lacked the secondary symptoms seen in survivors elsewhere (Reichard and Kunz 2009; present study). This suggests that these bats belong to remnant populations possessing behavioral and/or physiological traits that aid in surviving WNS. Our results from captive little brown myotis and free-ranging European *Myotis* species lead us to hypothesize that antibodies are not the mechanism behind such survival, but instead that high anti-*Pd* antibody titers in these bats are correlates of survival. It is also possible that the increased titers observed in northeastern populations of little brown myotis reflect greater exposure by living in areas of endemic *Pd* exposure, but this is not consistent with what was observed for other species in the same hibernacula, or with what was seen in European *Myotis*.

The correlation between anti-*Pd* titer and survival may be similar to that found in mice exposed to the fungal pathogen *Candida albicans*, where antibodies were found to be predictors of the ability of mice to survive infection once titers exceed a certain threshold, even though cell-mediated immune responses were the mechanism of successful host defense (Spellberg et al. 2008). Although monoclonal antibodies have been shown to have protective roles in immune-defense against fungal pathogens, evidence that naturally produced antibodies has a protective effect is lacking (Casadevall and Pirofski 2012a,b; Wüthrich et al. 2012).

In the case of WNS, the opportunity for antibodies to play a role in host defense is furthered hampered by the downregulation in immune function ubiquitously observed among mammalian hibernators (Bouma et al. 2010a). In rodents, for example, hibernation is associated with leukopenia (Bouma et al. 2010a,b, 2011), reductions in complement activity (Maniero 2002), and decreased antibody production (Burton and Reichman 1999; Bouma et al. 2013). Despite this reduction, however, hibernating woodchucks (*Marmota monax*) produce antibodies during hibernation, even when primary exposure occurs during hibernation (Beaudoin et al. 1969), and no decline in antibody responses to a T-cell-dependent antigen was observed in a study of hibernating 13-lined ground squirrels (*Ictidomys tridecemlineatus*) (Bouma et al. 2013). Similarly, we observed that little brown myotis have detectable levels of anti-*Pd* antibodies during winter, and in some cases exhibited relatively high titers.

Our results from free-ranging little brown myotis sampled in the winter and spring, and bats exposed to *Pd* in captivity, suggest that anti-*Pd* antibody levels peak shortly after emergence from hibernation. Thus, circulating antibodies against *Pd* in little brown myotis peak after bats have left their hibernacula, outside of which the fungus is unable to grow due to temperature restrictions on *Pd* growth (Verant et al. 2012). Other studies of WNS-affected bats suggest both the presence (Meteyer et al. 2012; Moore et al. 2013) and absence (Meteyer et al. 2009) of an inflammatory immune response to *Pd* during winter (see also the discussion in Brook and Dobson 2015). Although our results suggest that much of the immune response to *Pd* occurs in spring, consistent with the work of Meteyer et al. (2012), it is critical to note that we sampled visibly sick bats during winter, suggesting these bats were unlikely to survive. Thus, studies of little brown myotis populations surviving with WNS are needed to better assess circulating antibody during winter.

It is notable that several little brown myotis populations far outside the WNS-affected zone tested positive for anti-*Pd* antibodies. Little brown myotis must, therefore, produce antibodies that are cross-reactive with *Pd* and other pathogens. Cross-reactivity of antibodies to various fungal pathogens is not uncommon, with antibody binding often occurring at common molecular surface patterns shared across many species (Rappleye et al. 2007). Thus, antibodies that recognize these surface molecules – a common pattern recognized by antifungal antibodies (Wüthrich et al. 2012) – will recognize *Pd*. A great number of fungal species are present in bat hibernacula (Lorch et al. 2013), serving as possible sources of primary exposure to molecular patterns to which antibodies can be produced.

Not all species produced antibodies against *Pd*, however, and anti-*Pd* antibody titers varied significantly among species. These differences among species may be linked to differences in winter ecology or physiology. We predicted that big brown bats and Rafinesque's big-eared bats would exhibit higher antibody titers because these species have relatively low WNS mortality rates (Turner et al. 2011), arouse more frequently from hibernation (Brack and Twente 1985; Twente et al. 1985; Johnson et al. 2012), and because immune function (at least in rodents) is known to be rapidly restored during periodic arousals (Maniero 2002; Bouma et al. 2011). Contrary to this prediction, however, these species exhibited lower seroprevalence and titers than the smaller little brown myotis. Another small species with infrequent arousals and high WNS mortality, tri-colored bats (Brack and Twente 1985; Twente et al. 1985; Turner et al. 2011), had the lowest titers and seroprevalence of any North American species. Thus, no consistent relationship was observed between antibody titer and body size or winter arousal frequency. Differences in WNS susceptibility across North American species cannot be explained by differences in ability to mount a humoral immune response.

Antifungal immune responses are not always protective and antibodies, in particular, can be associated with immune pathology instead of protection. Protection depends on the type of T cells activated and cytokines produced. For example, immune responses dominated by the Th2 phenotype are frequently characterized as permissive or exacerbating the effects of fungal disease (Cenci et al. 1997; Jain et al. 2009; Haraguchi et al. 2010) while Th17 cells generally promote protection from fungal infection (Conti et al. 2009; Hernández-Santos and Gaffen 2012). Unlike the role of antifungal antibodies in defense, cell-mediated immune responses have well-known roles in clearing fungal infections in mammals (Spellberg et al. 2008; Hernández-Santos and Gaffen 2012). Given that we were unable to detect antibodies against *Pd* in greater mouse-eared myotis infected with *Pd* during winter, and *Pd* is associated with pathology but not mortality in this species (Pikula et al. 2012), we hypothesize that cell-mediated immune responses aid in host defense in this species. Furthermore, we hypothesize that cell-mediated immune responses are also involved in host defense in North American species with relatively low WNS mortality rates, such as big brown bats and Rafinesque's big-eared bats (Frank et al. 2014). In our study, these species exhibited significantly lower seroprevalence and titers of anti-*Pd* compared to little brown myotis in Kentucky, despite presence of *Pd* on the skin of all species. Although *Pd* was present and anti-*Pd* antibody titers were low in big brown bats and Rafinesque's big-eared bats, we observed no wing damage among individuals of these species in Kentucky.

This study provides strong evidence that antibody-mediated immunity is not the mechanism behind survival of European or North American bats infected with *Pd*. Thus, our finding that anti-*Pd* antibody titers can be increased in little brown myotis through vaccination must be considered in the context of this role of antibody-mediated immunity. In mice, immunization against fungal pathogens shows that immunization can confer protection if cell-mediated immune responses are the result of vaccination and that protection was also associated with high antibody titers (Spellberg et al. 2008). Thus, although antibody-mediated immune responses are not a mechanism for promoting survival of WNS, immunizing bats may still confer protection against *Pd* if cell-mediated immune responses also result.

In conclusion, our study on circulating antibodies against the fungus that causes WNS provides evidence that an antibody-mediated immune response is insufficient to explain survival of North American or European bats infected with *Pd*. Although antibodies against *Pd* are correlates of protection for little brown myotis*,* their presence cannot explain the survival of remnant populations of little brown myotis in the northeastern United States (as documented by Dobony et al. 2011 and Reichard et al. 2014). The low *Pd* loads and absence of secondary injuries (Reichard and Kunz 2009; Meteyer et al. 2012) we observed among little brown myotis in remnant populations of survivors in New York and Pennsylvania suggest that these populations are physiologically different from populations in Kentucky, where *Pd* has more recently been introduced. These results provide hope that little brown myotis are adapting to life with WNS.

## Data Accessibility

All data are included in the supplementary material.

## Supporting Information

**Table S1.** Summary of sampling effort and anti-*Pd* antibody titers for seven North American and two European bat species captured between 2012 and 2014, sorted by season and study location. **Table S2.** Comparison of anti-*Pd* antibody seroprevalence and titers for four bat species sampled during spring 2013 and 2014 at Mammoth Cave National Park, Kentucky, USA. **Table S3.** Comparison of anti-*Pd* antibody seroprevalence and titers for little brown myotis sampled during winter, spring, and summer 2013-2014 in Kentucky, USA. **Table S4.** Comparison of *Pd* loads detected on bat species captured Mammoth Cave National Park, Kentucky, during the spring of 2014. **Table S5.** Comparison of anti-*Pd* antibody seroprevalence and titers for five bat species sampled during spring 2013 and 2014 at Mammoth Cave National Park, Kentucky, USA. **Table S6.** Comparison of anti-*Pd* antibody seroprevalence and titers for little brown myotis sampled at various locations in the United States during spring 2013-2014. **Table S7.** Comparison of *Pd* loads detected on little brown myotis captured at different regions of the United States during 2013-2014. **Table S8.** Comparison of anti-*Pd* antibody seroprevalence and titers for three bat species sampled during hibernation (March 2012 and 2014).
