# Supplementary material for: Antibodies to Pseudogymnoascus destructans are not sufficient for protection against white-nose syndrome
Source: Ecol Evol. 2015 May 11;5(11):2203–14. doi: 10.1002/ece3.1502 (PMC4461422; doi:10.1002/ece3.1502)
Supplement: Supplementary file 1 [file ece30005-2203-sd1.pdf]

Table S1. Summary of sampling effort and anti-*Pd* antibody titers for seven North American and two European bat species captured between 2012 and 2014, sorted by season and study location. Titers are presented as medians followed by range in parentheses.

Year	Season	Location	Species	*n*	Titer
2012	Winter	Germany	*Myotis myotis*	14	0 (0-0)
2013	Spring	Kentucky	*Eptesicus fuscus*	19	20 (10-37)
			Lasiurus borealis	1	10
			Myotis lucifugus	21	32 (16-249)
			Myotis septentrionalis	20	13 (10-32)
			Perimyotis subflavus	19	10 (10-11)
		New York	*Myotis lucifugus*	17	54 (22-248)
		Finland	*Myotis daubentonii*	6	17 (10-61)
	Summer	Kentucky	*Corynorhinus rafinesquii*	20	10 (10-22)
			Eptesicus fuscus	20	13.5 (10-35)
			Lasiurus borealis	2	10 (10)
			Myotis lucifugus	20	16 (10-43)
			Myotis septentrionalis	17	10 (10-47)
			Perimyotis subflavus	4	10 (10)
		Finland	*Myotis daubentonii*	4	20 (10-31)
2014	Winter	Kentucky	*Myotis lucifugus*	20	11.5 (10-122)
			Perimyotis subflavus	16	10 (10-19)
	Spring	Kentucky	*Corynorhinus rafinesquii*	15	12 (10-51)
			Eptesicus fuscus	17	15 (10-208)

	Lasiurus borealis	3	19 (10-21)
	Myotis lucifugus	18	28 (10-319)
	Myotis septentrionalis	17	17 (10-34)
	Nycticeius humeralis	6	10 (10-18)
	Perimyotis subflavus	15	10 (10-14)
Pennsylvania	*Myotis lucifugus*	15	35 (20-171)
Montana	*Myotis lucifugus*	6	10 (10-33)

Table S2. Comparison of anti-*Pd* antibody seroprevalence and titers for four bat species sampling during spring 2013 and 2014 at Mammoth Cave National Park, Kentucky, USA. Data reported are median titers (range in parentheses) and the number of seropositive plasma samples (total sample size in parentheses), alongside Pearson chi-square values testing for difference in prevalence, and Wilcoxon Z testing for differences in median antibody titer, between years. No comparisons were statistically significant ($P > 0.05$).

Species	2013 Titer	Positive Samples	2014 Titer	Positive Samples	χ^2	*Z*
Eptesicus fuscus	20 (10-37)	11 (20)	15 (10-208)	12 (17)	0.963	-1.64
Myotis lucifugus	32 (16-249)	21 (21)	28 (10-319)	17 (18)	1.197	-0.80
Myotis septentrionalis	13 (10-32)	14 (20)	17 (10-34)	12 (17)	0.002	-1.27
Perimyotis subflavus	10 (10-11)	4 (19)	10 (10-14)	1 (15)	1.554	-1.10

Table S3. Comparison of anti-*Pd* antibody seroprevalence and titers for little brown myotis sampled during winter, spring, and summer 2013-2014 in Kentucky, USA. Titers are presented as medians followed by range in parentheses. For each measure, seasons not sharing common letters differed significantly ($P < 0.05$).

Season	*n*	Positive Samples	Median Titer
Winter	20	12 [a]	12 (10-122) [a]
Spring	39	38 [b]	32 (10-319) [b]
Summer	20	11 [a]	16 (10-43) [a]

Table S4. Comparison of *Pd* loads detected on bat species captured Mammoth Cave National Park, Kentucky, during the spring of 2014. Data are presented as median *Pd* loads (genomic equivalents) plus range in parentheses. Species not sharing common letters differed significantly ($P < 0.05$).

Species	*n*	*Pd* load
Myotis lucifugus	18	5535 (10-34,555) [a]
Myotis septentrionalis	17	10 (0-14925) [a, b]
Eptesicus fuscus	17	10 (0-5,699) [b]
Perimyotis subflavus	15	1157 (0-22,623) [c]
Corynorhinus rafinesquii	15	10 (0-1,527) [b]

Table S5. Comparison of anti-*Pd* antibody seroprevalence and titers for five species sampled during spring 2013 and 2014 at Mammoth Cave National Park, Kentucky, USA. Titers are presented as medians followed by range in parentheses. For each measure, species not sharing common letters differed significantly ($P < 0.05$).

Species	*n*	Positive Samples	Titer
Myotis lucifugus	39	39 [a]	32 (10-319) [a]
Myotis septentrionalis	37	31 [b]	14 (10-48) [b]
Eptesicus fuscus	36	33 [b]	18 (10-46) [b]
Corynorhinus rafinesquii	15	7 [b]	10 (10-162) [b]
Perimyotis subflavus	34	5 [c]	10 (10-12) [c]

Table S6. Comparison of anti-*Pd* antibody seroprevalence and titers for little brown myotis sampled at various locations in the United States during spring 2013-2014. Titers are presented as medians followed by range in parentheses. For each measure, seasons not sharing common letters differed significantly ($P < 0.05$).

Sampling Location	*n*	WNS Status	Positive Samples	Titer
Montana	6	Negative	2 [a]	10 (10-33) [a]
Kentucky	39	Positive since 2011	38 [b]	32 (10-319) [b]
Pennsylvania	15	Positive since 2008	15 [b]	35 (20-171) [b,c]
New York	17	Positive since 2006	22 [b]	54 (22-428) [c]

Table S7. Comparison of *Pd* loads detected on little brown bats captured at different regions of the United States during 2013-2014. Data are presented as median *Pd* loads (genomic equivalents) plus range in parentheses. States not sharing common letters differed significantly ($P < 0.05$).

State	*n*	*Pd* load
Kentucky	18	5535 (10-34,555) [a]
Pennsylvania	15	12 (0-508) [b]
New York	17	10 (0-440) [b]

Table S8. Comparison of anti-*Pd* antibody seroprevalence and titers for three species sampled during hibernation (March 2012 and 2014). Titers are presented as medians followed by range in parentheses. For each measure, species not sharing common letters differed significantly ($P < 0.05$).

Species	*n*	Sampling Location	Positive Samples	Titer
Myotis lucifugus	20	Kentucky, USA	12 [a]	12 (10-122) [a]
Perimyotis subflavus	16	Kentucky, USA	1 [b]	10 (10-19) [b]
Myotis myotis	14	Northern Bavaria, Germany	0 [b]	10 [b]
